# Comparison of MRI, [^18^F]FDG PET/CT, and ^99m^Tc-UBI 29-41 scintigraphy for postoperative spondylodiscitis—a prospective multicenter study

**DOI:** 10.1007/s00259-020-05109-x

**Published:** 2020-11-18

**Authors:** Diana Paez, Mike M. Sathekge, Hassan Douis, Francesco Giammarile, Shazia Fatima, Anil Dhal, Sunil K. Puri, Paola A. Erba, Elena Lazzeri, Rodolfo Ferrando, Paulo Almeida Filho, Vincent Peter Magboo, Olga Morozova, Rodolfo Núñez, Olivier Pellet, Giuliano Mariani

**Affiliations:** 1 Nuclear Medicine and Diagnostic Imaging Section, Division of Human Health, International Atomic Energy Agency, PO Box 100, A-1400 Vienna, Austria; 2 Nuclear Medicine Department, University of Pretoria & Steve Biko Academic Hospital, Pretoria, South Africa; 3 University Hospital Birmigham, NHS Foundation Trust, Birmingham, UK; 4 Department of Nuclear Medicine, Nuclear Medicine, Oncology & Radiotherapy Institute (NORI), Islamabad, Pakistan; 5 Department of Orthopaedics, Maulana Azad Medical College, New Delhi, India; 6 Department of Radiology, GB Pant Hospital, Maulana Azad Medical College, New Delhi, India; 7 Regional Centre of Nuclear Medicine, Department of Translational Research and Advanced Technologies in Medicine and Surgery, University of Pisa, Pisa, Italy; 8 Ferrari Ferrando-Paez Nuclear Medicine Clinic and Uruguayan Center of Molecular Imaging (CUDIM), Montevideo, Uruguay; 9 Nuclear Medicine Service, Real Hospital Português, Recife, Brazil; 10 University of the Philippines Manila, Manila, Philippines; 11 Excel Diagnostics and Nuclear Oncology Center, Houston, TX USA

**Keywords:** Spine surgery, Postoperative spine infection, Diagnostic imaging, MRI, [^18^F]FDG PET/CT, SPECT/CT with ^99m^Tc-UBI 29-41

## Abstract

**Purpose:**

Postoperative infection still constitutes an important complication of spine surgery, and the optimal imaging modality for diagnosing postoperative spine infection has not yet been established. The aim of this prospective multicenter study was to assess the diagnostic performance of three imaging modalities in patients with suspected postoperative spine infection: MRI, [^18^F]FDG PET/CT, and SPECT/CT with ^99m^Tc-UBI 29-41.

**Methods:**

Patients had to undergo at least 2 out of the 3 imaging modalities investigated. Sixty-three patients enrolled fulfilled such criteria and were included in the final analysis: 15 patients underwent all 3 imaging modalities, while 48 patients underwent at least 2 imaging modalities (MRI + PET/CT, MRI + SPECT/CT, or PET/CT + SPECT/CT). Final diagnosis of postoperative spinal infection was based either on biopsy or on follow-up for at least 6 months. The MRI, PET/CT, and SPECT/CT scans were read blindly by experts at designated core laboratories. Spine surgery included metallic implants in 46/63 patients (73%); postoperative spine infection was diagnosed in 30/63 patients (48%).

**Results:**

Significant discriminants between infection and no infection included fever (*P* = 0.041), discharge at the wound site (*P* < 0.0001), and elevated CRP (*P* = 0.042). There was no difference in the frequency of infection between patients who underwent surgery involving spinal implants versus those who did not. The diagnostic performances of MRI and [^18^F]FDG PET/CT analyzed as independent groups were equivalent, with values of the area under the ROC curve equal to 0.78 (95% CI: 0.64–0.92) and 0.80 (95% CI: 0.64–0.98), respectively. SPECT/CT with ^99m^Tc-UBI 29-41 yielded either unacceptably low sensitivity (44%) or unacceptably low specificity (41%) when adopting more or less stringent interpretation criteria. The best diagnostic performance was observed when combining the results of MRI with those of [^18^F]FDG PET/CT, with an area under the ROC curve equal to 0.938 (95% CI: 0.80–1.00).

**Conclusion:**

[^18^F]FDG PET/CT and MRI both possess equally satisfactory diagnostic performance in patients with suspected postoperative spine infection, the best diagnostic performance being obtained by combining MRI with [^18^F]FDG PET/CT. The diagnostic performance of SPECT/CT with ^99m^Tc-UBI 29-41 was suboptimal in the postoperative clinical setting explored with the present study.

**Supplementary Information:**

The online version contains supplementary material available at 10.1007/s00259-020-05109-x.

## Introduction

Postoperative infection rates following spinal surgery have been reported over a wide range from 1% up to 6–15% of patients—and even higher when utilizing metallic spinal implants [1, 2]. Although lower rates (1–4%) have more recently been reported [3–6], postoperative infection still constitutes an important complication of spine surgery that often requires hospitalization/reoperation, with increased healthcare costs [7].

Early clinical diagnosis of postoperative spinal infection remains challenging, since symptoms are often nonspecific and inflammatory markers remain abnormal early postsurgery. A combination of laboratory tests and imaging investigations follows clinical suspicion of postoperative spinal infection [8].

Although planar X-ray imaging may reveal periimplant lysis, reduced disc space, and/or bone destruction, it often fails to diagnose spondylodiscitis until 2–8 weeks after onset of symptoms. Computed tomography (CT) is relatively insensitive to diagnose early postoperative spinal infections, and is predominantly used for guiding biopsy.

Whereas magnetic resonance imaging (MRI) has high diagnostic accuracy for infection in the nonoperated spine [9], the diagnosis of spondylodiscitis is more challenging in the postoperative spine. The few MRI-based studies in postoperative spondylodiscitis are hampered by small sample sizes and are partially contradictory [10–13], thus leaving the role of MRI in the diagnosis of postoperative spondylodiscitis uncertain.

PET imaging with [^18^F]FDG for assessing infection/inflammation is growing [14], especially for spinal infection [8, 15] where the high interstitial pressure due to infection-related edema prevents efficient accumulation of radiolabeled cells at the infection site. Compared to CT and MRI, [^18^F]FDG PET/CT imaging provides the advantages of whole-body coverage to detect also unsuspected distant foci of infection, and lesser artifacts due to metallic implants.

In a recent multicenter retrospective study comparing the diagnostic accuracy of [^18^F]FDG PET/CT and MRI in 44 patients [16], the diagnostic performance of [^18^F]FDG PET/CT was consistently greater than that of MRI, although not reaching statistical significance: 86.4% sensitivity versus 66.7%, 81.5% specificity versus 75.0%, 79.2% positive predictive value versus 66.0%, 88.0% negative predictive value versus 75.0%. The authors concluded that a prospective study is needed to validate their preliminary results.

Regarding single-photon emitting agents to image infection independent from leukocyte binding, interest is rising on a ^99m^Tc-labeled fragment of the natural antimicrobial compound ubiquicidin, a 59-amino acid peptide part of innate immunity against infection [17]; its cationic 29-41 fragment (UBI 29-41) binds preferentially to the anionic microbial cell membrane [18]. Good diagnostic performance of ^99m^Tc-UBI 29-41 scintigraphy to identify bacterial infection has been reported in various conditions, including osteomyelitis [19–21].

In summary, although MRI is currently being perceived as the gold standard for diagnosing preoperative spinal infection, there remains a paucity of evidence supporting its role in the clinical scenario of suspected postoperative infection. The aim of this prospective study was to determine the diagnostic performance of MRI, scintigraphy with ^99m^Tc-UBI 29-41, and [^18^F]FDG PET/CT in patients with suspected postoperative spine infection.

## Patients and methods

This prospective multicenter Coordinated Research Project (CRP E13040), initiated by the Section of Nuclear Medicine and Diagnostic Imaging, Division of Human Health of the International Atomic Energy Agency (IAEA, Vienna, Austria), was conducted over a 4-year period from December 2013 to December 2017. Clinical follow-up continued for at least six additional months for those patients who did not undergo bacteriologic culture and/or diagnostic biopsy.

The protocol adopted for the study includes three imaging modalities (MRI, [^18^F]FDG PET/CT, and SPECT/CT with ^99m^Tc-UBI 29-41). Patients had to undergo at least two of such imaging modalities within a predefined time window to be included in final data analysis; time window was 2 weeks between MRI and PET/CT or SPECT/CT, and 1 week between PET/CT and SPECT/CT. A total of 63 patients fulfilled these criteria, as well as all the inclusion criteria listed further below.

In addition to the requirement of prior spinal surgery (with or without implantation of metallic spinal implants), the inclusion criteria were as follows:

1. Age of at least 18 years at the time of evaluation.
2. Clinical suspicion of postoperative spinal infection after spinal surgery.
3. Willingness to undergo the MR, PET/CT, and/or SPECT/CT scans.

To the purpose of the present study, the sole implantation of spinal devices, e.g., infusion pump for analgesic purposes, was not considered sufficient to classify patients as having undergone spinal surgery.

Consensus was developed among participant regarding the criteria to be taken into account for supporting the clinical suspicion of postoperative spinal infection. Such criteria were (1) fever (persisting beyond the 2nd postoperative day in case of early postoperative infection), (2) pain at the surgical site (especially of the throbbing type), (3) an inflamed, erythematous suture line, (4) discharge at the surgical wound, (5) raised inflammatory markers (ESR and CRP, especially with a further rising trend), and (6) elevated total leukocyte count. The presence of at least 4 of such 6 criteria would indicate a high clinical suspicion of postoperative infection (rising to very high suspicion in case of a dirty/purulent discharge versus a clear, serous discharge). On the other hand, the presence of 3 or less of such indicators (in isolation or combination) would translate into a low/medium suspicion of postoperative spine infection.

No time limitations were set regarding the period elapsed since spine surgery to onset of symptoms and diagnostic imaging, in order to include in the investigation both early and delayed postoperative infections. The exclusion criteria were as follows:

1. Pregnancy.
2. Known previous infection (discitis/osteomyelitis).
3. Known TB infection of the spine.
4. Known metastatic disease in the spine.
5. Poor compliance to MRI, PET/CT, and/or SPECT/CT examinations.
6. Absolute contraindications for MRI.

The final diagnosis of postoperative spinal infection was based on a CT-/fluoroscopic guided biopsy or open biopsy of the area of concern. When biopsy was not feasible, the diagnosis of spinal infection was based on bacterial culture of discharge at the wound site (if present), or on clinical follow-up and follow-up imaging for at least 6 months.

Patient specific data was collected on age, gender, site and type of previous spinal surgery, time interval between spinal surgery and suspected spinal infection, the presence of fever at presentation, discharge from wound site, presence/absence of a raised white cell count, ESR, and C-reactive protein.

Patients were recruited in the following eight countries: Argentina, Brazil, Colombia, India (two centers), Italy, Pakistan, South Africa, and Uruguay. In addition, blind reading of the MRI scans was performed at a center in the UK, and statistical analysis was performed in The Philippines.

The IAEA supported this study and helped recruit and qualify centers for the study. After approval of the study protocol by the internal IAEA Research Committee, clearance for each participating center was granted by its own local/national Ethics Committee. All patients gave written informed consent, and the study was performed in accordance with the Helsinki Declaration.

### Magnetic resonance imaging

Due to the multicenter nature of the study, patients were scanned on MRI-scanners from different manufacturers, but all of them at least 1.5 T. The recommended MRI-protocol included the following sequences: sagittal T1W1, axial T1WI, sagittal T2W1, axial T2WI, sagittal STIR-sequences, sagittal T1 FS W1 with contrast if applicable, and axial T1FS WI with contrast if applicable. Choice of other MRI parameters (TR, TE, matrix size, etc.) was left to the each participating center according to the specific MR scanner utilized for imaging.

### PET/CT imaging with [^18^F]FDG

[^18^F]FDG PET/CT scanning was performed in line with established international guidelines for infection imaging [8, 14]. All patients had fasted for at least 6 h, and their blood sugar was < 11 mMol/L when injecting [^18^F]FDG. The injected [^18^F]FDG activity was adapted to patients’ weight using standard formulas. Patients were kept warm in a dimly-lighted room with low ambient noise during an uptake period of 60 min. PET acquisition was performed in 3D mode from midthigh to vertex in a caudocranial direction, with 3 min per bed position. The CT portion of the scan was acquired as part of the hybrid imaging protocol for attenuation and localization purposes, minimizing in each patient the radiation exposure associated with the CT scan by reducing voltage and current intensity with respect to a fully diagnostic CT scan. Image processing was performed using ordered subset expectation maximization iterative reconstruction (OSEM, 4 iterations, 8 subsets) with a Gaussian filter applied at full-width at half-maximum of 5.0 mm. The fused [^18^F]FDG PET/CT images were displayed in axial, sagittal, and coronal slices.

### Imaging with ^99m^Tc-UBI 29-41

Authorization to use ^99m^Tc-UBI 29-41 in patients enrolled for the study was granted at the participating centers in Brazil, India, Pakistan, South Africa, and Uruguay. Kits for the preparation of ^99m^Tc-UBI 29-41 were purchased through the National Institute of Nuclear Research of Mexico (ININ, “Dr. Nabor Carrillo Flores” Nuclear Centre, Toluca S/N, La Marquesa, Ocoyoacac, Mexico). Details on ^99m^Tc-labeling of UBI 29-41 and quality control for free ^99m^Tc-Pertechnetate are given in “Supplemental material 1”. Radiochemical purity > 97% was required for administration to patients. ^99m^Tc-UBI 29-41 (approximately 740 MBq) was injected i.v. as a single bolus within a maximum of 30 min after radiolabeling

Scintigraphy was performed in all five centers using SPECT/CT dual detector gamma cameras equipped with low energy high-resolution collimators. Scanning included an initial blood pool imaging (5-min flow dynamic acquisition) of the area of interest, followed by a planar whole-body scan at 10-min postinjection. A whole body anterior and posterior planar image was also acquired at 30-min postinjection. SPECT/CT imaging centered over the region of interest in the spine was then performed, acquiring the SPECT images with 180° rotation for each detector head, 3° angle for each frame, 20 s per frame, and 128 × 128 pixel matrix. Finally, a whole body anterior and posterior planar 2–4 h delayed image was acquired.

SPECT images were reconstructed in the axial, coronal, and sagittal planes after iterative processing with OSEM iterative reconstruction. Similarly as for the [^18^F]FDG PET/CT scan, the CT portion of the scan was acquired minimizing in each patient the radiation exposure associated with the CT scan, by reducing voltage and current intensity with respect to a fully diagnostic CT scan. The fused SPECT/CT images were displayed in axial, sagittal, and coronal slices.

### Blind reading of scans at core laboratories

At each center enrolling patients in the protocol, DICOM files of the images obtained with each imaging modality were rendered anonymous and sent to a central repository managed by the Nuclear Medicine Department of the Ludwig-Maximilian University (Munich, Germany) for subsequent analysis/interpretation, as detailed here below.

Core laboratories for blinded image interpretation were established at the following participating institutions: University Hospital Birmigham, NHS Foundation Trust, Birmingham, UK (for the MRI findings); Regional Centre of Nuclear Medicine, Department of Translational Research and Advanced Technologies in Medicine and Surgery, University of Pisa, Pisa, Italy (for the SPECT/CT findings); and Nuclear Medicine Department, University of Pretoria & Steve Biko Academic Hospital, Pretoria, South Africa (for the [^18^F]FDG PET/CT findings). All the anonymized images were read blindly without knowledge of the imaging modalities or knowledge of the underlying diagnosis, adopting the following criteria for MRI, PET/CT, and SPECT/CT, respectively.

#### Interpretation of MRI images

All MRI images were analyzed by two musculoskeletal radiologists in consensus, who were blinded to the clinical diagnosis and laboratory findings. The diagnosis of spinal infection on MRI was based on the presence of a paravertebral, epidural, or discal fluid collection with rim enhancement. If such findings were absent, four out of the following six criteria had to be fulfilled for the diagnosis of postoperative spinal infection:

1. Bone marrow edema-like signal adjacent to the intervertebral disk (defined as bone marrow being hypointense on T1-weighted images and hyperintense on T2WI/STIR-sequences).
2. Bone marrow enhancement adjacent to the intervertebral disk.
3. Loss of the low signal intensity vertebral endplate on T1WI.
4. Hyperintensity of the disc on T2WI or STIR-images.
5. Disc enhancement after injection of gadolinium.
6. The presence of paravertebral or epidural inflammation (defined as increased signal intensity of the paravertebral or epidural tissues on T2WI or STIR-sequences).

#### Interpretation of [^18^F]FDG PET/CT images

All the [^18^F]FDG PET/CT scans were scored blindly at the central core laboratory by two expert readers not aware of the results of histology, microscopy, and/or clinical follow-up. Differences in interpretation were resolved by consensus between the readers at the same core laboratory; when agreement could not be reached, a third expert at another core laboratory contributed to clarify the findings. PET findings were scored as positive or negative based on visual interpretation using the semiquantitative score detailed below. Images were reviewed first without attenuation correction, then with attenuation correction using the CT transmission data.

The PET scans were evaluated searching for sites of abnormally increased [^18^F]FDG uptake greater than surrounding background and not due to normal organ uptake, according to the following five-point scale: 0 = no uptake; 1 = uptake < descending aortic blood pool; 2 = uptake ≈ blood pool; 3 = uptake < liver but > blood pool; 4 = uptake > liver.

Scores 3 and 4 were considered positive for infection. In case of equivocal interpretation based on the score described above, the scan was considered as positive if [^18^F]FDG uptake was detected in the volume of interest (discectomy, spinal fusion with or without implants) or in the volume of interest and in an adjacent volume. Faint [^18^F]FDG uptake limited to a volume adjacent to the area of interest was considered as negative for infection.

#### Interpretation of SPECT/CT images

The planar and SPECT/CT scans were scored blindly at the core laboratory by two expert readers who were not aware of the results of histology, microscopy, or clinical follow-up. As suggested by Akhtar et al. [19], a 4-point scale for uptake of ^99m^Tc-UBI 29-41 at the site of suspected infection was adopted, as follows: 0 = no uptake; 1 = uptake < liver; 2 = uptake ≥ liver; 3 = uptake > kidneys. In case of discrepant interpretation of the planar and SPECT/CT images, the hybrid component of the study was considered to constitute the correct criterion for interpreting the scan.

According to Akhtar et al. [19], score 2 was considered as the cutoff threshold for infection. Additional analyses assessed either score 1 or score 3 as possible thresholds for infection, to explore less stringent (score 1) and more stringent (score 3) criteria, respectively, for the diagnosis of infection.

### Statistical analysis

All data were collected in a structured database using SPSS statistics (version 20.0; IBM Corp.) and diagnostic value of MRI, [^18^F]FDG PET/CT, and ^99m^Tc-UBI 29-41 SPECT/CT was determined by calculating sensitivity, specificity, PPV, NPV, and overall diagnostic accuracy, with 95% confidence intervals (CI). The positive and negative likelihood ratios as well as the diagnostic odds ratios were calculated as well.

Statistical significance was tested using chi-square test and ROC analysis. A *P* value of less than 0.05 was considered significant. The statistical analysis was performed using SPSS version 20.0 (SPSS, Chicago, IL, USA).

## Results

As per December 2017, a total of 69 patients were recruited in the study; final data analysis is based on 63 patients, 6 cases being excluded either because the time window between the scans exceeded the predefined time frame, or because patients were lost to follow-up, and no final diagnosis of infection versus no infection was available. Based on the diagnostic parameters listed above, 30 out of the 63 patients (or 48%) were classified as having postoperative spinal infection at the time of enrolment in the study. Median time between spine surgery and onset of symptoms suggesting infection was 7 months, ranging from a minimum of one week to a maximum of 7 years. The main clinical and biochemistry features of the 63 patients included in the final analysis are summarized in Table 1. The most common patients’ complain, while elevated ESR and CRP were the most common signs; according to surgeons’ assessment, almost two-thirds of the patients were classified as having low/medium probability of infection, whereas one-third of patients were classified as high/very high probability of infection (see Table 1). Statistically significant discriminants between infection and no infection include the presence of fever (*P* = 0.041), discharge at the wound site (*P* < 0.0001), and elevated CRP (*P* = 0.042, serum CRP being 21.01 mg/L ± 5.28 SEM in patients with infection versus 6.09 mg/L ± 1.53 SEM in those without infection). The presence of back pain, elevated leukocyte count, and elevated ESR did not discriminate between infection and no infection. Infections rates were not different in patients who underwent surgery with implantation of metallic spinal hardware (20/44 or 45.5%) versus patients without implants (10/19 or 52.6%).

**Table 1:** Main demographic, clinical, and blood chemistry features of the 63 patients included in final analysis

Sex ratio (M:F)	36:27
Mean age (y)	53.8 ± 17.4
Age range (y)	18 – 84
Median age (y)	57
Spinal surgery with instrumentation	44/63 (69.8%)
Level of spinal surgery ^1^	
Cervical	9
Thoracic	18
Lumbar	51
Sacral	19
Clinical presentation raising the suspicion of postoperative spine infection ^2^	
Back pain	62/63 (98.4%)
Fever	28/63 (44.4%)
Discharge at wound site ^3^	12/63 (15.9%)
Blood chemistry findings	
Elevated leukocyte count	33/63 (52.4%)
Elevated ESR	42/63 (66.7%)
Elevated CRP	39/63 (61.9%)
Probability of infection according to surgeons’ assessment	
Low, medium	40/63 (63.5%)
High, very high	23/63 (36.5%
Final diagnosis based on biopsy	33/63 (51%)
Final diagnosis based on microbiological culture at surgical wound ^4^	7/63 (12%)
Final diagnosis based on follow-up	23/63 (37%)

(^1^) Possible surgery involving more than one level in different patients (e.g., cervico-thoracic, thoraco-lumbar, lumbo-sacral)

(^2^) More than one symptom possible in patients

(^3^) Discharge at wound site was purulent in 7 cases an serous in 5 cases

(^4^) Results of microbiological culture of discharge at wound site: *Staphilococcus aureus* in 3 patients (methicillin-resistant in 1 case); Gram-positive cocci in 1 patient; *Escherichia coli* in 1 patient; *Pseudomonas aeruginosa* in 1 patient; no bacterial growth in 1 patient

Table 2 reports the combination of imaging modalities employed in the group of 63 patients included in the final analysis: 16 patients underwent all three imaging modalities, while 48 patients underwent at least two imaging modalities (either MRI + PET/CT, MRI + SPECT/CT, or PET/CT + SPECT/CT). The time interval between onset of symptoms raising the suspicion of postoperative infection and imaging was shorter than 5 days in all patients, just the time required for formal enrolment in the study protocol.

**Table 2:** Different combinations of imaging modalities employed in the whole group of 63 patients included for final analysis

Combination of imaging modalities	Patient
MRI + [^18^F]FDG PET/CT	25
MRI + ^99m^Tc-UBI 29-41 SPECT/CT	22
MRI + [^18^F]FDG PET/CT + ^99m^Tc-UBI 29-41 SPECT/CT	16
[^18^F]FDG PET/CT **+** ^99m^Tc-UBI 29-41 SPECT/CT	17

**Fig. 1:**
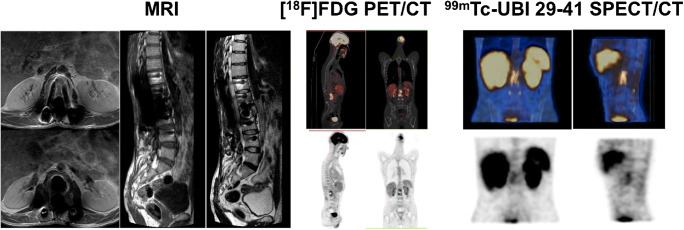
A 41-year-old man previously submitted to spinal fusion T12-L4 presented with lower back pain; ESR and serum CRP levels were markedly increased. Diagnosis of infection in this patient was based on biopsy. On MRI (left) the sagittal T1WI and T2WI of the thoracolumbar spine and axial T1WI with contrast of the lumbar spine demonstrate extensive posterior spinal implant extending from T12 to L4, which results in marked metal artifacts at the site of surgery. No MRI features to suggest spondylodiscitis can be identified. The [^18^F]FDG PET CT images (middle) show markedly increased tracer uptake, especially in the upper segments of the lumbar spine. The ^99m^Tc-UBI 29-41 SPECT/CT images (right) show markedly increased tracer uptake in the same segments exhibiting increased [^18^F]FDG uptake. Final diagnosis of postoperative spine infection was established in this patient on the basis of biopsy findings

**Fig. 2:**
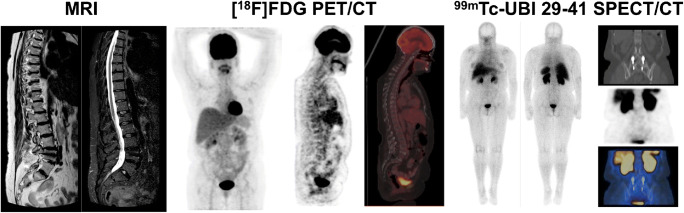
A 52-year-old woman previously submitted to lumbar surgery L4-S1 presented with sensory loss in the L2-L3 region; serum CRP was mildly increased, ESR was normal. On MRI (left) the sagittal T2WI and STIR images of the thoracolumbar spine demonstrate posterior spinal implant at L4 with no MRI features to suggest postoperative spondylodiscitis. No increased tracer uptake was noted on [^18^F]FDG PET CT images (middle) nor on ^99m^Tc-UBI 29-41 SPECT/CT (right). Final diagnosis of this patient at the final 7-month follow-up was “no postoperative spine infection”

Examples of imaging in two patients who underwent all three imaging modalities (MRI, [^18^F]FDG PET/CT, and SPECT/CT with ^99m^Tc-UBI 29-41) are reported in Figs. 1 and 2.

Table 3 reports the parameters of diagnostic performance obtained with each of the three imaging modalities employed during the study considered separately with reference to their ability to distinguish postsurgical infection versus no infection of the spine. All results of statistical analysis refer to the whole patients’ population, regardless of the use or not of implants during spine surgery; in fact, the majority of patients (46 out of 63) had had surgery involving spinal implants, thus leaving a population of only 17 patients without implants—too small for reliable statistical analysis.

**Table 3:** Comparative diagnostic performances of the three imaging modalities employed during the study, considered as separate procedures (with 95% confidence intervals within brackets)

	MRI	PET/CT	SPECT/CT	SPECT/CT
	(*n* = 48)	(*n* = 44)	(*n* = 49; score ≥ 2)	(*n* = 49; score ≥ 1)
Sensitivity	0.71 (0.51–0.87)	0.63 (0.35–0.85)	0.44 (0.20–0.70)	1.00 (0.79–1.00)
Specificity	0.83 (0.59–0.96)	0.89 (0.67–0.99)	0.83 (0.64–0.94)	0.41 (0.24–0.61)
Positive predictive value	0.87 (0.70–0.95)	0.83 (0.56–0.95)	0.58 (0.35–0.79)	0.48 (0.41–0.56)
Negative predictive value	0.65 (0.50–0.78)	0.74 (0.60–0.84)	0.73 (0.63–0.81)	1.00
Diagnostic accuracy	0.76 (0.61–0.87)	0.77 (0.70–0.90)	0.69 (0.53–0.82)	0.62 (0.47–0.76)
Positive likelihood ratio	4.29 (1.49–12.36)	5.94 (1.52–23.24)	2.54 (0.96–6.71)	1.71 (1.26–2.32)
Negative likelihood ratio	0.34 (0.18–0.64)	0.42 (0.22–0.80)	0.68 (0.43–1.08)	0.00
Diagnostic odds ratio	12.5 (2.83–55.26)	14.17 (2.39–84.07)	3.73 (0.94–14.84)	23.57 (1.29–430.82)
Area under ROC curve *	0.78 (0.64–0.92)	0.80 (0.64–0.98)	0.663 (0.46–0.81)	0.707 (0.56–0.86)

(*) Diagnostic performance according to area under the ROC curve:

Area > 0.9 = highly accurate diagnostic test

Area between 0.7–0.9 = fairly good diagnostic test

Area between 0.5–0.7 = poor diagnostic test

Area < 0.5 = worthless diagnostic test

Among the three imaging modalities employed, [^18^F]FDG PET/CT yielded an area under the ROC curve (AUC) equal to 0.8, which translates into a “fairly useful” diagnostic test, corresponding to 63% sensitivity and 89% specificity (see Table 3). The MRI findings yielded an AUC equal to 0.78, which translates into a “fairly useful” diagnostic test, corresponding to 71% sensitivity and 83% specificity (see Table 3). On cross-comparison, no statistically significant difference was found between the diagnostic performance of [^18^F]FDG PET/CT and of MRI.

As to SPECT/CT with ^99m^Tc-UBI 29-41, considering scores > 2 as positive for infection [19] yielded an area under the ROC curve equal to 0.633; this translates into a “poor” diagnostic test, corresponding to satisfactory specificity (83%), associated however with a disappointingly low sensitivity (44%) (see Table 3). A lower threshold for positivity (scores > 1, less stringent interpretation criteria) yielded an area under the ROC curve equal to 0.707, corresponding to a “fairly useful” diagnostic test. However, the extremely high sensitivity so obtained (100%) was achieved at the expense of a very low specificity (41.4%) (see Table 3). On the other hand, raising the threshold of the ^99m^Tc-UBI 29-41 score to 3 yielded very high specificity (96.2%), associated however with an extremely low sensitivity (12.5%); the corresponding area under the ROC curve was 0.543, which corresponds to a “poor” diagnostic test (data not shown in Table 3).

Statistical analysis of the different pair combinations of imaging modalities (MRI combined with [^18^F]FDG PET/CT, MRI combined with ^99m^Tc-UBI 29-41 SPECT/CT, and [^18^F]FDG PET/CT combined with ^99m^Tc-UBI 29-41 SPECT/CT) considered only the concordant cases for each combination, that is, 16/25 patients for the MRI + PET/CT combination, 22/30 patients for the MRI + SPECT/CT combination, and 17/19 patients for the PET/CT + SPECT/CT combination. ROC curve analysis identified MRI + [^18^F]FDG PET/CT as the best diagnostic combination (AUC = 0.938, a “highly accurate” diagnostic test), followed by the two radionuclide-based scans (AUC = 0.833, a “fairly good” diagnostic test). MRI combined with ^99m^Tc-UBI 29-41 SPECT/CT yielded a significantly lower diagnostic accuracy (AUC = 0.589, a “poor” diagnostic test).

The results obtained in the 16 patients who underwent all three imaging modalities combined (MRI + [^18^F]FDG PET/CT + SPECT/CT) showed an extremely high specificity (100%), associated however with low sensitivity (50%); the positive predictive was 100%, overall accuracy was 99.88%, and the AUC under the ROC curve was 0.75.

Analysis of the discordant cases within each pair combination is shown in “Supplemental file 2”. Out of the total 19 patients in whom discordant results were found between each pair of imaging modalities for the three possible pair combinations, the MRI findings were correct in 12 cases (63%), the [^18^F]FDG PET/CT findings were correct in 4 cases (21%), and the ^99m^Tc-UBI 29-41 SPECT/CT findings were correct in 3 cases (16%). All 12 patients in whom MRI was correct had infection. Out of the 7 patients in whom either [^18^F]FDG PET/CT or SPECT/CT was correct, 3 patients had infection (all of them identified by [^18^F]FDG PET/CT), and 4 did not have infection (3 of them being correctly identified by SPECT/CT with ^99m^Tc-UBI 29-41, 1 by [^18^F]FDG PET/CT).

## Discussion

The economic costs and health care utilization associated with postoperative spine infection are very high, averaging about USD 38,000 per patient (but up to USD 78,900 in some cases) [22]. Diagnostic tests enabling early diagnosis of postoperative spine infection promote timely initiation of appropriate treatment, therefore reducing such high treatment-related costs.

The results obtained in this study provide ground for several considerations. In general, our data confirm that clinical judgement alone is not reliable enough to correctly identify postoperative spine infection, which was confirmed in only 30 out of the 63 patients in whom it had been suspected on clinical grounds (< 50% of the cases). The only discriminant factors with statistical significance as to infection versus no infection were discharge at the wound site, fever, and elevated CRP.

Concerning each imaging modality separately, in our patients’ population the diagnostic performances of [^18^F]FDG PET/CT and MRI were approximately equivalent, with only marginal differences in sensitivity, specificity, and the associated diagnostic parameters. The values given in Table 3 for the area under the ROC curve correspond to the best reasonable compromise between sensitivity and specificity. Depending on the clinical scenario where either [^18^F]FDG PET/CT and/or MRI is employed, one might want to maximize sensitivity (thus making the scan especially useful to rule out infection) or specificity (thus making the scan especially useful to ascertain with certainty the presence of infection—yet being aware that some cases with infection will be missed). Either of these two goals can reasonably be achieved by adopting less or more stringent criteria when interpreting the scan as positive for infection.

The diagnostic performances of MRI and [^18^F]FDG PET/CT in suspected vertebral infection for patients not previously submitted to spinal surgery have been reported to be virtually equivalent in terms of sensitivity, specificity, positive predictive value, and negative predictive value [23–25]. The results obtained in the 48 patients of this study evaluated with MRI demonstrate that this imaging modality is a fairly good diagnostic test for spine infection also in the postoperative setting, provided that well-defined interpretation criteria are adopted.

It is reasonable to assume that, in this scenario, radionuclide imaging based on functional/metabolic evaluation of the local changes induced by infection can provide useful diagnostic information to complement the morphologic and structural information provided by MRI. [^18^F]FDG PET/CT data from 315 intervertebral disc levels (based however on only 9 patients without prior spine surgery) showed the SUV_max_ of infected disc levels to range between 3 and 12.7, with an optimal threshold of 4.2 for infection [23]. These results are hardly transferable directly to our study population of patients with suspected postoperative spine infection. Therefore, when interpreting the [^18^F]FDG PET/CT scans we did not rely on SUV, also because this parameter has not been proven to discriminate patients with from those without infection in general, including suspected spine infection [8].

Overall, the results obtained in our prospective study reach similar conclusions as in a recent retrospective study that compared the diagnostic performances of [^18^F]FDG PET/CT and MRI in patients with suspected postoperative spine infection following surgery with hardware implant [16]. The parameters of diagnostic performance of the two imaging modalities are also very similar in the two studies, although slightly better in a consistent manner in our study than in the study by Follenfant et al. [16]. For easier comparison, Table 4 lists the main diagnostic parameters obtained in the two studies (sensitivity, specificity, positive, and negative predictive values). Altogether, these two studies support the notion that imaging with either MRI and/or [^18^F]FDG PET/CT provide crucial information to establish a correct diagnosis in patients with suspected postoperative spine infection, including patients either with or without implants. Moreover, another important conclusion reached in our prospective study, previously not identified, is that MRI and [^18^F]FDG PET/CT provide somewhat complementary information, as demonstrated by the optimal diagnostic performance obtained when combining the two imaging modalities, with an AUC equal to 0.938 at ROC analysis (see Table 4). This results in excellent confidence for confirming or excluding postoperative spine infection. In this regard, the growing diffusion of PET/MR instrumentation raises interesting issues on the potential benefits of hybrid [^18^F]FDG PET/MR imaging in patients with suspected infection as a novel “single-shop stop”. Although the benefits of this imaging approach have been explored in patients with as variety of benign musculoskeletal conditions including infection in general [26], no sufficient data are so far available regarding patients with suspected postoperative spine infection.

**Table 4:** Comparison of the main diagnostic parameters obtained in the present study with those reported by Follenfant et al. [16] in a similar patients’ population. Follenfant et al. analyzed their data as a head-to-head comparison of the MRI findings with the [^18^F]FDG PET/CT findings in the same patients. In the present study, the diagnostic performance of the two imaging modalities was analyzed as separate groups of patients independently from one another imaging modality

	This study	Ref. [16] (*n* = 44)
MRI		
Sensitivity	0.71	0.67
Specificity	0.83	0.75
Positive predictive value	0.87	0.66
Negative predictive value	0.65	0.75
[^18^F]FDG PET/CT		
Sensitivity	0.63	0.86
Specificity	0.89	0.82
Positive predictive value	0.83	0.79
Negative predictive value	0.74	0.88

SPECT/CT imaging with ^99m^Tc-UBI 29-41 yielded somewhat puzzling results in our patients’ population. Adopting the threshold for positivity indicated by Akhtar et al. [19] yielded good specificity (83%), associated however with unacceptably low sensitivity (44%); this resulted in an overall AUC under the ROC curve equal to 0.663, which indicates a poor diagnostic test. On the other hand, lowering the threshold to score 1 (less stringent criteria for positivity) did improve sensitivity—but resulted in an unacceptably low specificity (41%). The suboptimal diagnostic performance of SPECT/CT with ^99m^Tc-UBI 29-41 is carried along when this scan is combined with MRI (see above). A somewhat intermediate result is obtained by combining the two radionuclide-based imaging modalities, PET/CT and SPECT/CT. It is unlikely that such low sensitivity of ^99m^Tc-UBI 29-41 for clear-cut positive scans (score 2) is due to the lower resolution properties of SPECT/CT imaging versus MRI or PET/CT imaging. The possibility must therefore be considered that the localization properties of ^99m^Tc-UBI 29-41 per se are not always optimal. In this regard, quality control of ^99m^Tc-UBI 29-41 after radiolabeling and before injection into patients relies solely on free ^99m^Tc-pertechnetate—a quality control step favorably passed at all participating centers. Nonetheless, there might be other physicochemical factors altering the binding properties of ^99m^Tc-UBI 29-41 in such a way that cannot be ascertained with the current quality control procedure.

**Fig. 3:**
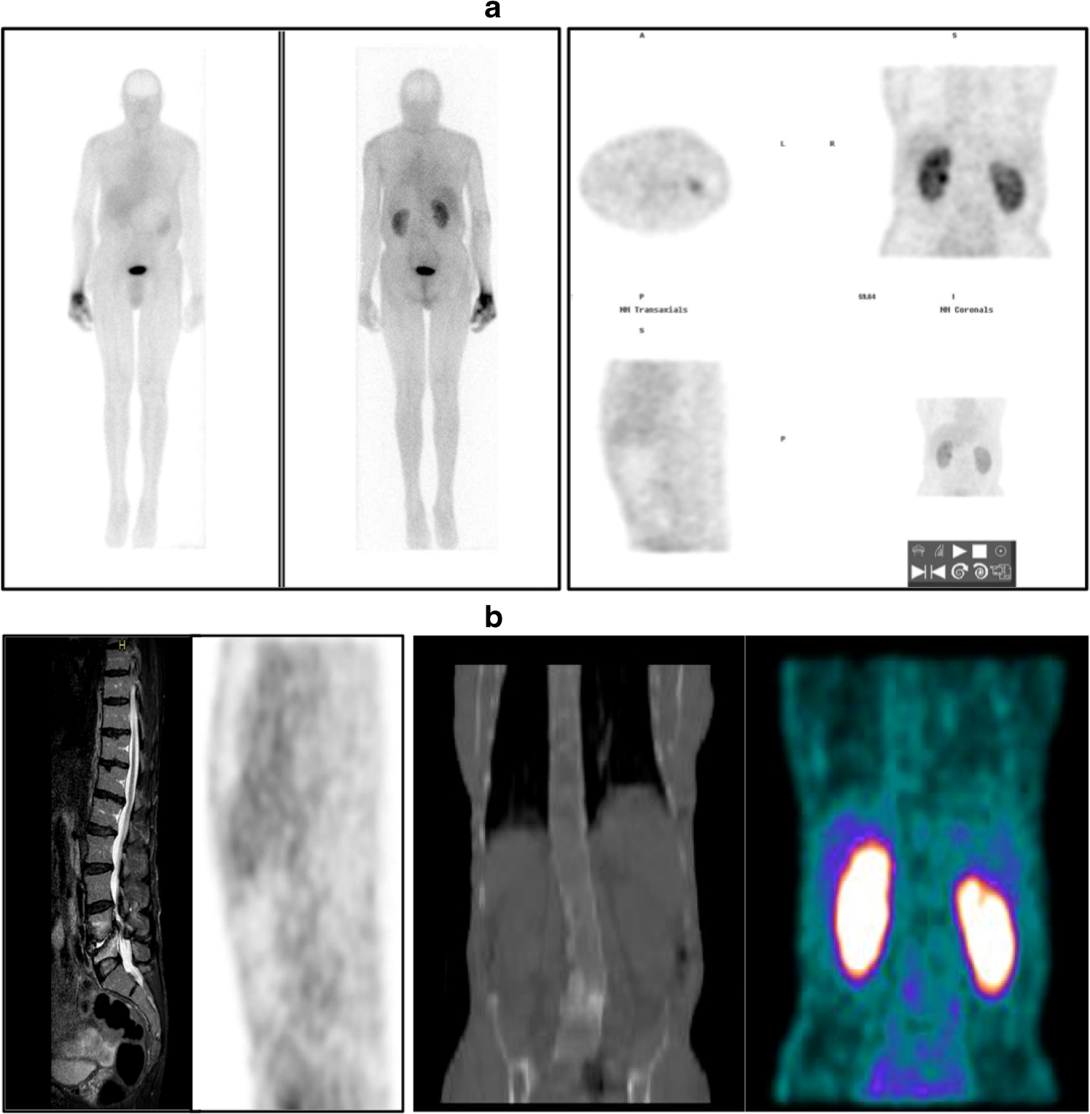
A 57-year-old man presented with back pain, fever, and raised CRP level (21.32 mg/L) about 12 months after being submitted to lumbar surgery L3-L5. (**a**): anterior and posterior whole-body views recorded about 30 min after injection of ^99m^Tc-UBI 29-41 (left panel), and selected tomographic sections of the emission SPECT/CT scan acquired immediately thereafter (right panel). (**b**), from left to right: sagittal MRI section, sagittal SPECT section, coronal CT section, and coronal fused SPECT/CT section. MRI demonstrates enhancement of end plates and disc indicating active infection. Whereas, no clear foci of abnormal ^99m^Tc-UBI 29-41 uptake can be detected in either the planar or SPECT/CT images; physiologic tracer uptake in the liver of this patient appears lower than in the patients of Figs. 1 and 2 because the images are displayed with lower contrast. Following prolonged high-dose antibiotic therapy all symptoms disappeared and the serum CRP level returned to normal. This case was therefore classified as a false negative ^99m^Tc-UBI 29-41 SPECT/CT scan

In conclusion, [^18^F]FDG PET/CT and MRI both possess equally satisfactory diagnostic performance in patients with suspected postoperative spine infection, the best diagnostic performance being obtained by combining MRI with [^18^F]FDG PET/CT. Instead, the diagnostic performance of SPECT/CT imaging with ^99m^Tc-UBI 29-41 resulted to be suboptimal for diagnosing spine infection, at least in the postoperative clinical setting explored with the present study (see example of a false-negative ^99m^Tc-UBI 29-41 study in Fig. 3). When compared to other infection tracers such as radiolabeled antibiotics (no longer commercially available), ^99m^Tc-UBI 29-41 exhibits much lower target-to-background ratios in infected lesions. The question remains unanswered whether this is due to its lower binding specificity or to a lower number of molecules bound per bacteria [18]. Furthermore, discrepant indications derive from published studies regarding the optimal time window for imaging infection with ^99m^Tc-UBI 29-41, whether at 30 min or 60 min postinjection. Although tracer accumulation at the infection site seems to peak around 30 min [21], interpretation of imaging at 60 min might be facilitated by the lower blood pool activity at this later time. There is an ongoing debate about the optimal imaging time for best target-to-background ratios, which should be settled on the basis of larger clinical experience in different centers.

**Fig. 4:**
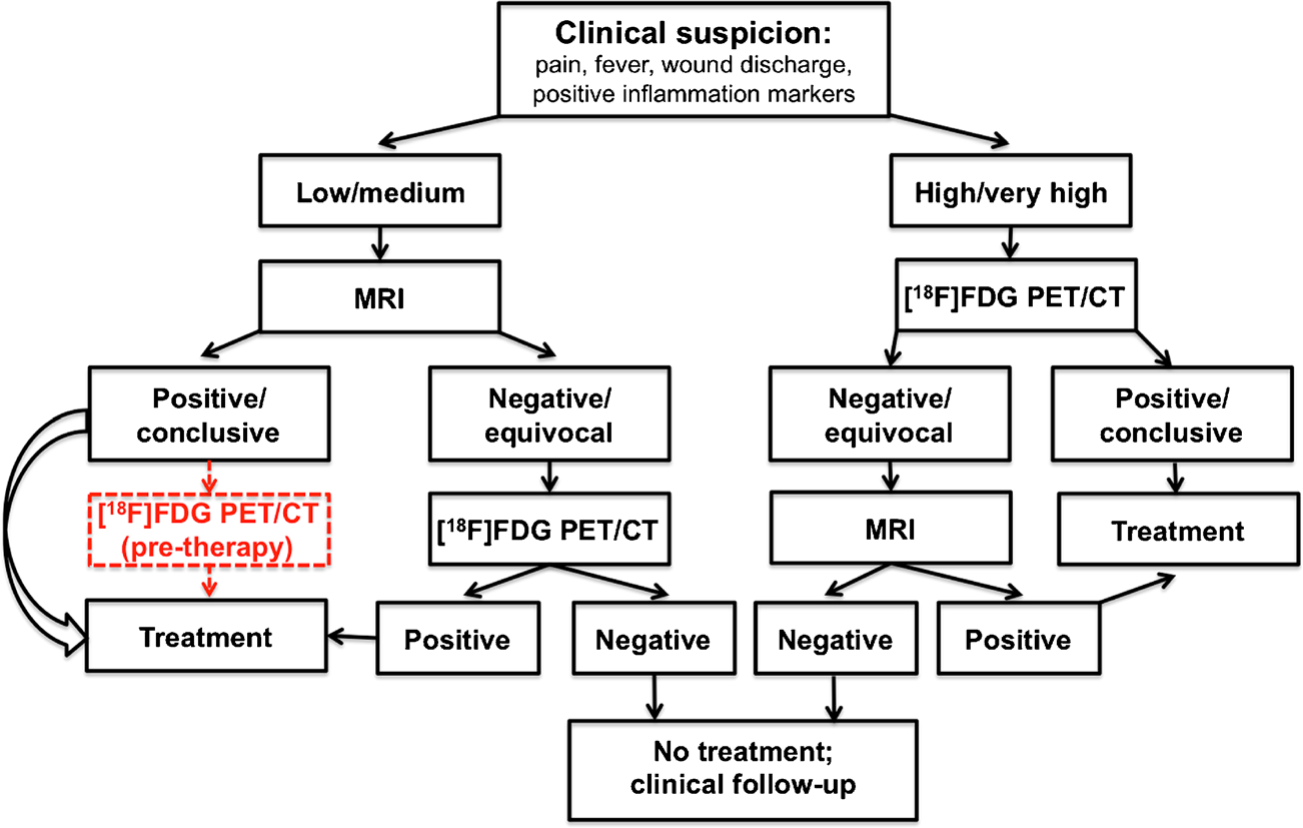
Proposed algorithm for diagnostic imaging in patients with suspected postoperative spine infection; other confirmatory tests (biopsy, microbiological culture of discharge at wound site) are not indicated in this flow diagram—which only concerns imaging. Because of suboptimal diagnostic performance and limited commercial availability, the use of ^99m^Tc-UBI 29-41 SPECT/CT imaging is not included in this diagnostic algorithm, which is expected to have more general validity and applicability. Clinical suspicion prompts diagnostic imaging for confirmation and assessment of the extent/severity of infection. Although rather insensitive for infection (especially early post-surgery), plain X-ray (not indicated in the diagram) can occasionally be used to rule out gross skeletal changes such as displacement of bone segments and/or metallic implants. This diagram is based on 2 basic assumptions: 1) both MRI and [^18^F]FDG PET/CT are readily available in an ideal hospital environment; 2) the trend for better sensitivity of MRI and better specificity of [^18^F]FDG PET/CT observed in this study is firmly established by additional controlled clinical trials. In the left side of the diagram, [^18^F]FDG PET/CT is introduced after the diagnosis of postoperative spine infection has been established by MRI, as a possible option for a baseline scan before starting therapy, considering better suitability of functional metabolic imaging ([^18^F]FDG PET/CT) rather than MRI for assessing response to therapy [ref 8]. In case of positively established postoperative spine infection, treatment will depend on imaging findings and on clinical ground (e.g., presence of neurological signs/symptoms, sepsis, stability problems of the spine, etc.). In case of limited access to MRI and to PET/CT imaging, the relatively high specificity of ^99m^Tc-UBI 29-41 SPECT/CT makes this imaging modality a valuable option to confirm infection in case of a positive scan

Based on the overall results obtained in this study, it can safely be speculated that a practical diagnostic algorithm in patients with suspected postoperative spine infection includes either MRI or [^18^F]FDG PET/CT, whichever is accessible within the shortest time window since initial clinical suspicion of infection. Although optimal diagnostic performance is achieved by combining these two imaging modalities, further investigations should aim at assessing the cost-effectiveness of performing separately both examinations systematically in any given patients with suspected postoperative infection of the spine. Although the results obtained in this study showed no statistically significant differences in the diagnostic performance parameters between MRI and [^18^F]FDG PET/CT, there are nevertheless slight differences that might in principle favor the use of one imaging modality over the other in particular clinical scenarios. Considering that a highly specific diagnostic test correctly identifies the nonaffected individuals (because of very low false-positive results) and considering the parameters of diagnostic performance reported in Table 3, in case of high/very high clinical suspicion of infection [^18^F]FDG PET/CT might be preferred as first-line imaging over MRI. On the other hand, a highly sensitive diagnostic test correctly identifies the affected individuals (because of very low false-negative results); therefore, MRI might be preferred as first-line imaging over [^18^F]FDG PET/CT to exclude the presence of disease in case of low/medium clinical suspicion of infection. Based on these considerations, a tentative algorithm might be envisaged for the complementary use of MRI and [^18^F]FDG PET/CT in patients with suspected postoperative spine infection, as shown diagrammatically in Fig. 4. This algorithm is based on the assumptions that both MRI and [^18^F]FDG PET/CT are readily available in an ideal hospital environment and that the trend for better sensitivity of MRI and better specificity of [^18^F]FDG PET/CT observed in this study is firmly established by additional controlled clinical trials. On the other hand, in case MRI is not readily accessible and no PET/CT equipment is available in the local Nuclear Medicine center, the relatively high specificity of ^99m^Tc-UBI 29-41 SPECT/CT makes this imaging modality a valuable option to confirm infection in case of a positive scan.

## Supplementary Information

ESM 1 (DOCX 121 kb)

ESM 2 (DOCX 54 kb)
