# Supplementary material for: Comparison of MRI, [18F]FDG PET/CT, and 99mTc-UBI 29-41 scintigraphy for postoperative spondylodiscitis—a prospective multicenter study
Source: Eur J Nucl Med Mol Imaging. 2020 Nov 18;48(6):1864–75. doi: 10.1007/s00259-020-05109-x (PMC8113215; doi:10.1007/s00259-020-05109-x)
Supplement: Supplementary file 1 — (DOCX 121 kb) [file 259_2020_5109_MOESM1_ESM.docx]

**Supplemental material 1: ^99m^Tc-labeling of UBI 29-41**

^99m^Tc-pertechnetate freshly eluted from a ^99^Mo/^99m^Tc generator was used for radiolabeling, strictly following the manufacturer’s instructions. Each lyophilized kit contains 25 µg of UBI 29-41 and 12.5 µg of stannous chloride as the reducing agent. A maximum volume of 1 mL of the ^99^Mo/^99m^Tc generator’s eluate containing at least 740 MBq of ^99m^Tc-pertechnetate is added to the vial; the labelling reaction is completed by adding 40 µL of 0.1 M NaOH and incubating for 15 min at room temperature. Routine quality control of the radiopharmaceutical was performed by ITLC on silica gel using 0.9% NaCl as the solvent; in this system, ^99m^Tc-Tc-UBI 29-41 does not migrate from the origin, while ^99m^Tc-pertechnetate migrates with the solvent’s front. Radiochemical purity >97% was the strict requirement for administration to patients. ^99m^Tc-UBI 29-41 (approximately 740 MBq) was injected i.v. as a single bolus within a maximum of 30 min after radiolabeling.
