# Supplementary material for: Comparison of MRI, [18F]FDG PET/CT, and 99mTc-UBI 29-41 scintigraphy for postoperative spondylodiscitis—a prospective multicenter study
Source: Eur J Nucl Med Mol Imaging. 2020 Nov 18;48(6):1864–75. doi: 10.1007/s00259-020-05109-x (PMC8113215; doi:10.1007/s00259-020-05109-x)
Supplement: Supplementary file 2 — (DOCX 54 kb) [file 259_2020_5109_MOESM2_ESM.docx]

Discordant cases for the three pair combinations of diagnostic investigations considered in this study

Diagnostic Discordant Of which correct, #

combination cases, #

MRI + PET/CT 9 MRI: 6; PET/CT: 3

MRI + SPECT/CT 8 MRI: 6; SPECT/CT: 2

PET/CT + SPECT/CT 2 PET: 1; SPECT/CT: 1
